# A comparative study of glycoproteomes in androgen-sensitive and -independent prostate cancer cell lines

**DOI:** 10.1007/s11010-013-1857-6

**Published:** 2013-10-09

**Authors:** Anna Drabik, Dorota Ciołczyk-Wierzbicka, Joanna Dulińska-Litewka, Anna Bodzoń-Kułakowska, Piotr Suder, Jerzy Silberring, Piotr Laidler

**Affiliations:** 1 Department of Biochemistry and Neurobiology, AGH University of Science and Technology, Mickiewicza 30 Ave, 30-059 Krakow, Poland; 2 Chair of Medical Biochemistry, Jagiellonian University Medical College, Kopernika 7 Str, 31-034 Krakow, Poland; 3 Center of Polymer and Carbon Materials, Polish Academy of Sciences, Sowinskiego 5 Str, 44-121 Gliwice, Poland

**Keywords:** Proteome, Lectin affinity chromatography, Prostate cancer, Cell lines, DU145, LNCaP

## Abstract

Prostate cancer is one of the most common malignancies in men and is predicted to be the second leading cause of cancer-related deaths. After 6–18 months, hormone ablation treatment results in androgen-independent growth of cancer cells, metastasis and progression. The mechanism of androgen-independent growth of prostatic carcinoma cells is still unknown. Identification of factors that facilitate the transition from androgen-dependent to independent states is crucial in designing future diagnostics and medication strategies. To understand the biochemical meaning of hormone dependency deprivation, glycoproteins enriched profiles were compared between DU145 (hormone non-responding) and LNCaP (hormone responding) prostate cancer cells. These results allow for anticipation on the important role of glycosylation in malignant transformation. Both Tn antigen and complex antennary *N*-oligosaccharides were recognized. Their occurrence might be involved in the development and progression of tumor, and failure of hormone ablation therapy. Among identified proteins in androgen-sensitive cells nucleolin (P19338) was found that is widely described as apoptosis inhibitor, and also transporter of molecules from the membrane to the cytoplasm or nucleus. In addition, 14-3-3 protein family (P27348, P31946, P61981, P63104, P62258, Q04917, and P31947) was investigated across available databases as it forms stable complexes with glycoproteins. Our studies indicate that isoforms: sigma and eta were found in androgen-dependent prostate cancer cells, while other isoforms were present in androgen non-responding cells. 14-3-3 binding partners are involved in cancer pathogenesis. These findings may contribute to a better understanding of prostate cancer tumorigenesis and to a more efficient prognosis and individual therapy in a future. However, it still remains to be revealed how important those changes are for androgen dependency loss in prostate cancer patients carried out on clinically relevant populations.

## Introduction

**Fig. 1:**
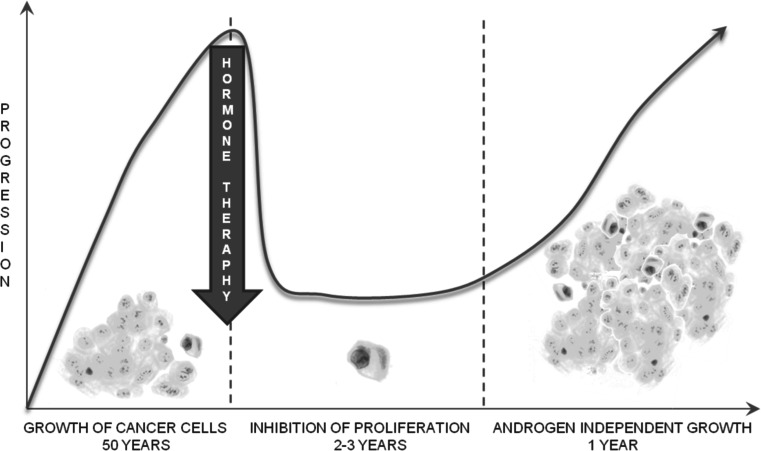
Performance of hormone therapy

Patients with advanced prostate cancer (PCa) initially benefit from androgen ablation therapy, which leads to the temporary tumor remission due to apoptosis of the androgen-sensitive tumor cells [1]. However, recurrence of androgen-independent tumor is inevitable for most patients and renders the conventional hormone therapy ineffective [Fig. 1]. Despite the extensive knowledge we currently have, the PCa mechanisms of the loss of androgen dependency by cancer cells that ultimately leads to the formation of metastases, is not recognized yet. Understanding the mechanism and identification of factors that facilitate the transition from androgen-dependent to independent state is crucial in designing future diagnostics and medication strategies. In the present study, we revealed lectin-enriched proteome changes in the androgen dependency loss between androgen-responding and androgen-nonresponding prostate cancer cell lines. The rationale to focus on glycoproteome is based on the fact that 30 of 38 proteins on the list of biomarkers, proteins involved in disease formation, currently used in clinical diagnostic are known to be glycosylated [2]. Aberrant protein glycosylation may be a result of various factors, including inflammation or cancer development. These changes result in abnormal alterations of biological functions, protein folding, adhesion, metastasis, and molecular recognition. The site(s) of protein glycosylation and the structure of the oligosaccharides are altered during initiation or progression of the disease; however, we have focused on analysis of glycosylated subproteome for the reason that affinity reagents reduce sample complexity, and also enrich reliability of the results in biologically and clinically important information.

Glycoproteomic studies are complicated by the micro- and macro-heterogeneity of glycoproteins in comparison to their non-glycosylated forms. Macro-heterogeneity is related to variability in the number of potential sites glycosylated in the same protein, whereas micro-heterogeneity is represented by the possibility to carry a variety of glycan structures at the same glycosylation site. Nature gives the full advantage of enormous diversity of glycans expressed in all living organisms by creation of lectins able to recognize discrete glycans that mediate specific physiological or pathological processes.

There are many known glycoprotein enrichment strategies, including hydrazine chemistry, titanium dioxide, enzymatic method, and lectin technique. However, only lectin affinity chromatography (LAC) allows detecting target glycans on specific protein carriers, and is routinely utilized methodology designed to concentrate glycoproteins [3]. Therefore, to facilitate isolation of glycoproteins from the total cell extract, the LAC technique was applied. The highly abundant proteins often do not possess affinity for lectins. For that reason, lectins may act as enriching factors for cancer-related aberrant species that may further be validated as potential cancer biomarkers. To measure the changes in protein patterns with the specific glycan structure in prostate cancer cells, we proposed a lectin affinity-based mass spectrometry method.

Low specificity and efficiency of lectins has been discussed in numerous studies [4–6]. Those limitations are the result of insufficient binding affinity, leading to poor sensitivity in analytical assays, and the lack of availability glycan-binding reagents for less studied structures. However, impressive achievements in MS technologies and instrumentation, also miniaturization benefits in increased sensitivity of proteomic investigations, and resulted in minimizing these drawbacks.

Abnormal glycosylation, such as, e.g., increased glycan size and extra branching of glycan chains with oversialylation and fucosylation, is closely associated with cancer progression. The presence of characteristic glycans including Tn antigens, and tri- and tetra-antennary *N*-glycans on the protein surface in healthy and malignant cells may be valuable for understanding pathological mechanisms of cancer progression, resistance to treatment, and for identifying specific cancer biomarkers [7]. Therefore, for the purpose of these studies we have selected both VVL (*Vicia Villosa*) and *Phaseolus vulgaris* agglutinin (PHA-L) lectins. VVL recognizes preferentially terminal *N*-acetylgalactosamine residue characteristic for the Tn antigen, whereas PHA-L binds to antennary *N*-oligosaccharides. The presence of glycosylation sites was determined by a simple method that utilizes the Swiss-Prot database and the Mascot search engine [8].

It was confirmed by the three biological, and two technical replicates, that the presented approach provides reproducible results with precision sufficient to distinguish differences in protein profiles between analyzed samples. Selected cell lines are characterized by different properties in terms of androgen dependency. DU145 cell line was derived from brain metastasis, and is an example of the cells found in patients who do not respond to hormonal treatment, mostly in the terminal state [9]. LNCaP cell line served as a model of tumor in patients who respond to androgen ablation therapy [10]. The cell lines model provides an additional advantage, as the amount of sample required for MS analysis is not limited, what makes cell lysates suitable for extensive fractionation. Furthermore, cell cultures allow for enrichment of proteins that are present at higher concentrations than in the patients’ serum or plasma.

## Materials and methods

**Fig. 2:**
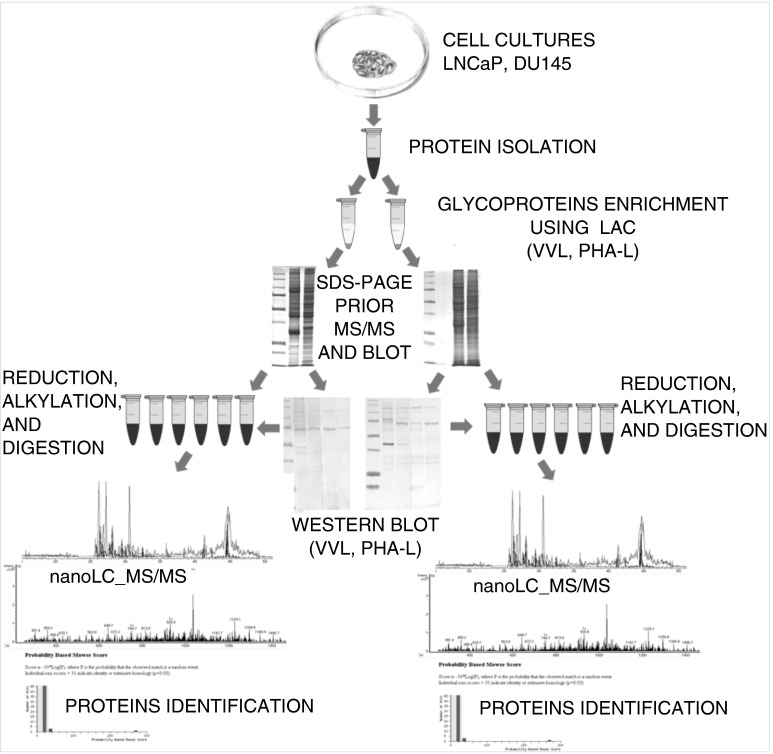
Proteomic strategy for identification of glycoproteins

DU145 (androgen-insensitive) and LNCaP (androgen-sensitive) human prostate cancer cell lines were obtained from the American Type Culture Collection (USA). Cells were cultured in the RPMI-1640 medium (Sigma-Aldrich, Poland) supplemented with 10 % heat-inactivated fetal bovine serum (Gibco, Poland), 1 % l-glutamine, 100 U/ml penicillin, 100 μg/ml streptomycin at 37 °C in a humidified atmosphere of 5 % CO_2_ [9, 10]. For the analysis, LNCaP and DU145 cell cultures were rinsed with PBS and homogenized three times on ice by sonication (5 s each) in 700 μl sample buffer consisting of 50 mM Tris/HCl pH 7.5 supplemented with 1 mM EDTA and 7 μl of proteinases inhibitor cocktail (Sigma-Aldrich, Poland). The homogenate was left on ice with 1 % Triton X-100 and 7 μl 3 % protamine sulfate (1 h), and then centrifuged at 16,000 × g for 1 h at 4 °C (Ultracentrifuge L7-65 Beckman, USA). Protein concentration was determined in supernatants with the aid of a Bradford assay kit (Sigma-Aldrich, Poland). An efficient technique for glycoprotein identification in prostate cancer cells characterized with different androgen dependency states was developed. Combination of modern methods involved: glycoproteins isolation using LAC, glycoproteins’ separation based on the one-dimensional sodium dodecyl sulfate polyacrylamide gel electrophoresis (SDS-PAGE), specific lectin–protein interactions verification using Western-blotting, separation of peptides and identification utilizing capillary chromatography combined with tandem mass spectrometry (nanoLC-MS/MS) (Fig. 2).

### Isolation of glycoproteins

Homogenates containing 1.2 mg of protein were incubated with either VVL or PHA-L lectins linked to agarose (Vector Lab, Poland) for 1 h at room temperature in the presence of 70 μl 10 mM HEPES buffer pH 7.5 with 0.15 M NaCl (Sigma-Aldrich, Poland), followed by 16 h incubation at 4 °C. Subsequently, suspensions were centrifuged at 22,000 × g for 5 min. at 4 °C. Precipitates were washed three times with PBS (Sigma-Aldrich, Poland), followed by incubation at 100 °C for 10 min in 50 μl of a buffer 50 mM Tris/HCl pH 6.8, 5 % BME, 2 % SDS, 10 % glycerol (Bio-Rad, Poland), 1 mM EDTA (Sigma-Aldrich, Poland), and centrifuged at 22,000 × g for 5 min at room temperature. Seventy microliters of supernatant were collected to the siliconized Costar tubes.

### SDS-PAGE

One-dimensional electrophoresis was performed. Thirty microliters of glycoprotein containing supernatant per lane was separated by 10 % SDS-PAGE, according to Laemmli protocol [11]. One part of the gel was stained with Coomassie Brilliant Blue G (CBB G) (Sigma-Aldrich, Poland) prior nanoLC-MS/MS analysis, and the second was electro transferred onto the PVDF membrane (Roche, Poland) to confirm the type of protein-lectin interactions.

### Western blot analysis

**Fig. 3:**
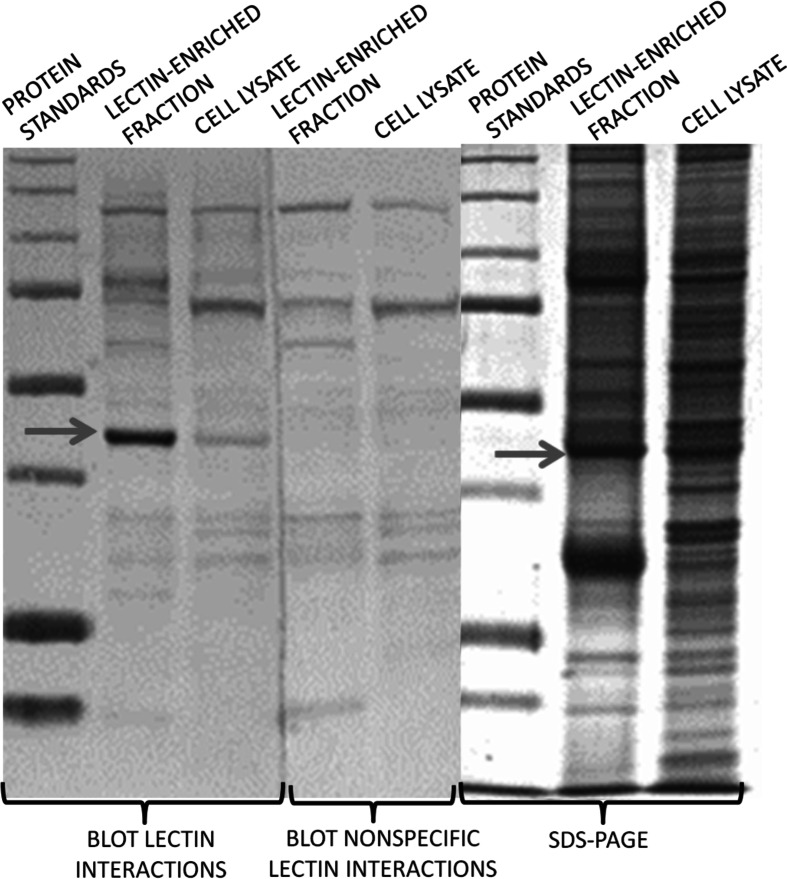
Example of lectin–protein interactions blots (LNCaP proteins precipitated and detected with VVL lectin)

The presence of specific glycan epitopes, Tn antigens and highly complex antennary *N*-oligosaccharides was confirmed by VVL and PHA-L lectins, respectively. The blotted membranes were treated with casein solution as a blocking agent for 1 h at room temperature, washed three times with tris buffered saline (TBS) buffer (Sigma-Aldrich, Poland), and subsequently incubated with VVL or PHA-L lectin for 2 h, according to manufacturer’s recommendations. After extensive washes, the blots were incubated with alkaline phosphatase substrate kit II (Vector Lab, Poland). Simultaneously, negative controls were included in the presence of lectins blocked with 0.2 M GalNAc (Sigma-Aldrich, Poland), and, respectively, 0.1 M CH_3_COOH (Sigma-Aldrich, Poland), that are responsible for blocking glycan-binding sites on the receptor surface. The nonspecific interactions were not considered for further investigations (Fig. 3).

### Extraction of peptides

The spots were excised from the CBB-stained gel with a scalpel, chopped into cubes, rinsed with water, and transferred into siliconized tubes. CBB stain was removed with 100 mM NH_4_HCO_3_ and an equal volume of acetonitrile was added after 10–15 min. Then, gel pieces were treated with 100 % acetonitrile and re-swollen in 12 ng/μL trypsin (Promega, USA) in 50 mM NH_4_HCO_3_ on ice for 45 min. The supernatants, which were not absorbed by gel particles, were removed, and gel pieces were immersed in 50 mM NH_4_HCO_3_ and incubated overnight at 37 °C. After completion of digestion, the supernatants were transferred into another tube, followed by addition of 50 mM NH_4_HCO_3_, and after 10–15 min, an equal volume of acetonitrile was added. The samples were incubated under shaking at 37 °C for 30 min. Extraction of peptides was repeated twice with 5 % formic acid (v/v) in acetonitrile, and combined extracts were evaporated to dryness in a vacuum centrifuge.

### Capillary chromatography combined with tandem mass spectrometry

Dried samples were prepared for nanoLC-MS/MS by dissolving them in 11 μl 0.1 % trifluoroacetic acid (TFA) (Sigma-Aldrich, Poland). The nanoLC-MS/MS analysis, used to separate the digests, was performed with the ultimate LC micro chromatography system (LC Packings/Dionex, USA). The separation was made on a capillary column filled with the PepMap reversed-phase material (15 cm long, 75 μm ID, C18, 2–3 μm particle size, and 100 Å pore size, LC Packings/Dionex, USA). The gradient was formed using 0.1 % HCOOH in 98:2 (v/v) water/acetonitrile solution (solvent A) and 0.1 % HCOOH in 20:80 (v/v) water/acetonitrile solution (solvent B), and it was delivered at a flow rate of 400 μL/min. The system was controlled by the Chromeleon software (Dionex, USA). A gradient was produced from 2 to 50 % B in 30 min and up to 90 % B at 35 min, then kept until 45 min, and again reduced to 2 % until 55 min. The chromatographic system was coupled directly to the Esquire 3,000 quadrupole ion-trap mass spectrometer (Bruker Daltonics, Germany) using the home-made “black-dust” nanoelectrospray emitter [12]. The instrument operated in a positive-ion mode. During analysis, two most intense peaks (threshold above 100,000) in the range 450–1,800 *m/z* were automatically fragmented in data-dependent acquisition mode. The acquired spectra were analyzed using the Bruker Data Analysis 4.0 software and were identified using Mascot 2.3.01 algorithm against the Swiss-Prot/TrEMBL sequence database 57.15 (515203 sequences; 181334896 residues). Search parameters were set as follows: taxonomy: human, modification: carbamidomethyl (fixed), up to 1 missed cleavage, peptide charges +1, +2, and +3, mass tolerance 0.8 Da for precursor mass, and 0.6 Da for fragment mass. The probability score evaluated by the software was used as criterion for correct identification, and also for comparison of molecular weight based on SDS-PAGE. Proteins with more than two-fragmented peptides detected were considered, and an additional criterion was a Mowse score above 40. Forty-nine of the androgen-independent cancer cell candidates were identified using capillary liquid chromatography combined with mass spectrometry based on these criteria (Table 1).

**Table 1:** Biomarker candidates of androgen dependency loss

Protein name	Protein ID	Number of identified peptides (based on MS/MS spectra)	Average score	Standard deviation	Present antigen
TLN1_HUMAN talin-1	Q9Y490	9	437	53	Antennary *N*-glycan
ITA9_HUMAN integrin α-9 precursor	Q13797	7	359	64	Tn
1433T_HUMAN 14-3-3 protein θ	P27348	8	397	51	Tn
1433B_HUMAN 14-3-3 protein β/α	P31946	5	285	46	Tn
1433G_HUMAN 14-3-3 protein γ	P61981	8	275	62	Tn
1433Z_HUMAN 14-3-3 protein ζ/δ	P63104	6	212	54	Tn
TBB4_HUMAN tubulin β-4 chain	Q13509	10	417	61	Tn
TBB1_HUMAN tubulin β-1 chain	Q9H4B7	7	391	59	Antennary *N*-glycan
MYH10_HUMAN myosin-10	P35580	11	326	73	Tn/antennary *N*-glycan
ACTZ_HUMAN α-centractin (centractin)	P61163	6	237	62	Tn
ACTC_HUMAN actin, α cardiac muscle	P68032	5	178	73	Tn
EF1A2_HUMAN elongation factor 1-α 2	Q05639	4	183	56	Tn
EF1B_HUMAN elongation factor 1-β	P24534	7	283	49	Antennary *N*-glycan
EF1G_HUMAN elongation factor 1-γ	P26641	6	219	65	Tn
COFA1_HUMAN collagen α-1(XV) chain precursor	P39059	6	290	54	Tn
CO5A2_HUMAN collagen α-2(V) chain	P05997	4	146	69	Tn
AT1A2_HUMAN sodium/potassium-transporting ATPase α-2	P50993	4	119	76	Tn
ATPO_HUMAN ATP synthase subunit O	P48047	5	89	36	Tn
VDAC3_HUMAN voltage-dependent anion-selective channel protein 3	Q9Y277	4	164	55	Tn
G6PI_HUMAN glucose-6-phosphate isomerase	P06744	5	183	63	Tn
PGM2_HUMAN phosphoglucomutase-2	Q96G03	6	192	68	Tn
KCRB_HUMAN creatine kinase B-type	P12277	6	117	53	Tn/antennary *N*-glycan
VDP_HUMAN general vesicular transport factor p115	O60763	5	192	49	Tn
EEA1_HUMAN early endosome antigen 1	Q15075	5	162	62	Antennary *N*-glycan
ROA1_HUMAN heterogeneous nuclear ribonucleoprotein A1	P09651	7	183	69	Antennary *N*-glycan
RU2A_HUMAN U2 small nuclear ribonucleoprotein A’	P09661	5	127	49	Tn
HNRH1_HUMAN heterogeneous nuclear ribonucleoprotein H	P31943	6	182	37	Tn
HNRPF_HUMAN heterogeneous nuclear ribonucleoprotein F	P52597	7	172	53	Tn
RL7_HUMAN 60S ribosomal protein L7	P18124	5	128	59	Tn
NUCL_HUMAN nucleolin	P19338	8	371	33	Tn
RL13_HUMAN 60S ribosomal protein L13	P26373	6	219	58	Tn
IF39_HUMAN eukaryotic translation initiation factor 3 subunit 9	P55884	4	163	38	Tn
RT07_HUMAN 28S ribosomal protein S7	Q9Y2R9	4	149	49	Tn
YBOX1_HUMAN nuclease-sensitive element-binding protein 1	P67809	6	238	40	Tn
UGDH_HUMAN UDP-glucose 6-dehydrogenase	O60701	4	217	58	Tn
ACADV_HUMAN very long-chain specific acyl-CoA dehydrogenase	P49748	5	173	53	Antennary *N*-glycan
ASPH_HUMAN aspartyl/asparaginyl beta-hydroxylase	Q12797	4	115	46	Tn
NQO1_HUMAN NAD(P)H dehydrogenase (quinone) 1	P15559	3	93	38	Antennary *N*-glycan
PKHA5_HUMAN pleckstrin homology domain-containing family A member 5	Q9HAU0	4	72	39	Tn
NPC1_HUMAN niemann-pick C1 protein	O15118	5	81	53	Antennary *N*-glycan
IQGA1_HUMAN ras GTPase-activating-like protein IQGAP1	P46940	3	148	58	Tn/antennary *N*-glycan
RAB8A_HUMAN ras-related protein Rab-8A	P61006	4	219	64	Tn
RAB8B_HUMAN ras-related protein Rab-8B	Q92930	6	246	38	Tn
RAN_HUMAN GTP-binding nuclear protein Ran	P62826	4	173	46	Tn
PSMD5_HUMAN 26S proteasome non-ATPase regulatory subunit 5	Q16401	3	116	58	Tn
IMB3_HUMAN importin β-3	O00410	6	82	47	Tn
PHB_HUMAN prohibitin	P35232	7	412	46	Tn
COMT_HUMAN catechol *O*-methyltransferase	P21964	4	184	39	Tn
TGM2_HUMAN protein-glutamine γ-glutamyltransferase 2	P21980	3	94	64	Antennary *N*-glycan

Average score was calculated based on three biological and two technical replicates

### Database searching

**Fig. 4:**
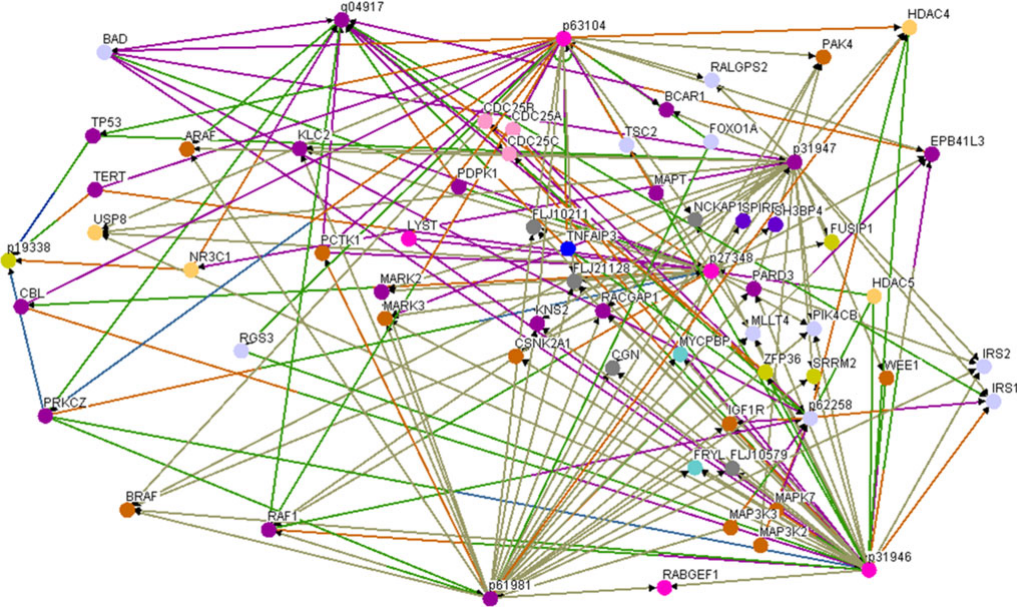
Interaction network of identified proteins visualized by Osprey Platform (BioGRID Database version 3.2.96)

In addition, all the identified proteins were searched against UniProtKB, Osprey and Panther Database to establish their molecular functions and interactions (Fig. 4). Numerous interaction nodes with oncogenes and proteins described as contributing directly to cancer progression were found.

## Results and discussion

Based on the available database information proteins contributing to cancer progression have been confirmed, therefore they can be the key to understand the mechanism of hormone dependency loss and might lead to the development of new treatment methods. Our special concern was dedicated to glycosylated nucleolin (P19338) and 14-3-3 protein family bounded with glycoproteins (P27348, P31946, P61981, P63104, P62258, Q04917, P31947), as these molecules are frequently reported to be involved in a variety of cellular processes, including tumorigenesis (Fig. 4).

Nucleolin carrying Tn antigen was found to be expressed in DU145 cells, and at the same time, deficient in LNCaP cells. In spite of its major nuclear localization, nucleolin is also known to shuttle between the nucleus and the cytoplasm, and during this trafficking it controls the organization of nuclear chromatin, RNA, DNA and ribosomes [13]. It is commonly known, that surface nucleolin is glycosylated and that *N*-glycosylation is crucial for its expression on the cell surface [14]. The presence of nucleolin on the cell surface seems to be an important factor in androgen dependency, since the hormone-sensitive cells do not contain this protein. This may suggest that the hormone-refractory cells evolved mechanism of blocking the extension of O-GalNAc, resulting in formation of incomplete glycans. Nucleolin is abundant in proliferating cancerous cells, and high levels of nucleolin expression are related to poor clinical prognosis [14–19]. In fact, with the increase of malignancy in patients, the raise of nucleolin levels present in cytoplasmic and membrane fractions is observed [17, 18]. In addition, P-selectin binds tumor cell surface nucleolin, but not nucleolin expressed in the cytoplasm or nucleolus, what may suggest a mechanism linking nucleolin to P-selectin-induced signal transduction pathways that regulate cell adhesion through activation of the α5β1 integrin. Because tyrosine kinase activity is important for the P-selectin-mediated nucleolin/PI3K interaction, tyrosine-phosphorylated nucleolin might participate in PI3K activation [19]. Furthermore, introduction of the anti-cancer aptamers that specifically bind to nucleolin resulted in inhibition of nucleolin function and cancer cell growth in vitro and in vivo [20]. We have previously performed studies on human melanoma cell lines and have shown that nucleolin may act as a marker of tumor progression, as its synthesis is correlated with increased cell proliferation [14]. Indirect immunofluorescence staining and laser scanning confocal microscopy were used to detect the presence of nucleolin in nucleolus, cytoplasm, and on the cell surface of human prostate cells. In contrast to nuclear nucleolin, the surface-expressed and cytoplasmic nucleolins exhibited Tn antigen, which was identified by simultaneous immunofluorescence staining and VVL-positive glycoproteins in confocal microscopy [14]. In conclusion, many questions remain to be resolved concerning the expression of nucleolin at the surface of cells and its trafficking, also in relation to the involvement of glycosylation in these processes. Further studies on the expression levels and translocation of nucleolin to the cell surface will certainly provide new insights into the mechanisms of androgen dependency loss, cancer development and progression.

The 14-3-3 proteins family involved in a growing number of cell biology processes, including modulation of cellular signaling pathways, cell death, cell cycle, and cytoskeletal dynamics was found to be differentially regulated according to different isoforms [21]. There are seven mammalian 14-3-3 family members (β, γ, ε, σ, ξ, τ, η) that are reported to be differently expressed between cell types and a variety of tissues [22]. During this study we have found two 14-3-3 isoforms: sigma (σ) and eta (η) among proteins characteristic for androgen-responding prostate cancer cells in VVL-enriched fractions as a result of interactions with series of glycoproteins, simultaneously absent in androgen-independent cells. These isoforms may have an impact on the loss of androgen dependency in prostate cancer cells, and may serve as an indicator of cell status. Possible mechanism might involve the recently described interactions with exonuclease 1 (Q9UQ84), where they function as apoptosis inducers, following DNA damage [23]. Epsilon isoform was identified in both cell types, and isolated by both VVL and PHA-Lectins. Finally, β, γ, ξ, and τ isoforms were found in VVL-precipitated fractions from DU145 cell homogenate. The principal regulatory mechanisms responsible for controlling the cellular levels of different 14-3-3 isoforms are still poorly understood. However, the sigma and eta isoforms are often described as tumor suppressors and their expression is up-regulated in cancer recurrent, while the remaining isoforms can work as potential oncogenes [24]. The main efforts on 14-3-3 biology in humans have been focused on their possible interactions and modifications of the enzymes functions that are of crucial importance in metabolic regulation [25, 26]. Presently, only a small fraction of 14-3-3 family have been thoroughly analyzed, despite the fact that there is a growing evidence for several hundred various binding partners [27]. In cancer progression, a key property of metastatic cells is the ability to migrate, and the first step in cell migration is modification of actin cytoskeleton. There are a number of proteins involved in actin remodeling that have been identified as 14-3-3 binding partners [28]. There is also an increasing evidence on the role of 14-3-3 as a transporter of binding partners to the cell membrane; however, further investigations are required to exploit the potential of the 14-3-3 proteins as drug targets.

Protein function is not only determined by the amino acid sequence, but also by various posttranslational modifications (PTM’s) that alter its biological role and affect formation of complexes with other molecules. Pathological changes influence the synthesis and metabolism of proteins and also modify interactions between them. This is particularly important in tumor growth and proliferation where the cells escape many control mechanisms. Serum derived from cancer patients typically contains complex protein patterns, often indirectly related to cancer disease. We believe that identification of the reliable cancer biomarkers in such complex mixture is a difficult task due to many ambiguities associated with detailed and reproducible analysis of serum proteins [29]. For instance, based on the gene expression studies on glycotransferases, only 1 % of the glycoprotein population is altered [2]. Therefore, we designed our experiments with culture cells, where glycoproteins are at much more abundant level, biological material is more homogenous and reproducible. In the view of the fact, that it is very challenging to generate a specific antibody against particular glycan structure, we employed lectins and their binding affinities toward sugar moieties for enrichment of samples. Lectins and carbohydrates are linked by a number of weak, non-covalent interactions. Their binding sites tend to be of a relatively low affinity, although they can exhibit high specificity. In addition, the lectin-binding specificity is determined by the amino acid residues that bind the glycan. Protein–protein interactions are considered to be much stronger than the binding of lectins to glycans; therefore the presence of non-glycosylated proteins in LAC-enriched fractions is well explicable.

Process of hormone sensitivity deprivation in prostate tumors leads to a multi-step cascade of cellular events, by which cancer cells escape control mechanisms and leave the original tumor mass to establish new colonies at distant sites in the body. To understand this mechanism, changes in glycoprotein-enriched profiles present as a result of androgen dependency loss were studied among proteins characteristic for the DU145 and LNCaP cells. The obtained results suggest that cell surface nucleolin, which is implicated in cell proliferation, tumor cell growth and angiogenesis, is relocated within the cell to the membrane, in addition to Tn antigen attachment [14]. Moreover, 14-3-3 protein isoforms σ and η seem to control key activities that may result in metabolic alteration and, as a consequence, may lead to cancer development [24, 25]. Both 14-3-3 isoforms η and σ significantly stimulate human exonuclease 1 activity, indicating that these regulatory proteins exert a common regulatory impact on hEXO1 [21]. Co-transfection of 14-3-3 leads to the enhanced tumorigenesis. Altogether these and our data anticipate that the cell tends to minimize proliferation of tumor-related pathways by lowering the level of 14-3-3 η and σ.

Described proteins may affect androgen administration, and might be involved in the development and progression of tumor, but also may lead to the failure of hormone ablation therapy.

The results of this study revealed that glycoprotein-enriched profile changes might serve as the putative prognostic marker that allows for differentiation between patients with PCa in the group responding to hormonal therapy and those who do not exert any positive effects. Therefore, our findings may have substantial impact, helping to target those individuals who urgently need radical intervention, while avoiding pharmacological overtreatment or incorrect diagnosis. The discovery of changes in proteome for tumor staging and monitoring PCa would greatly facilitate our understanding of the mechanism of androgen dependency loss.

## Abbreviations

BME: Beta mercaptoethanol
CBB: Coomassie Brilliant Blue
EDTA: Ethylenediaminetetraacetic acid
FDA: Food and drug administration
HEPES: 4(2-hydroxyethyl)-1-piperazineethanesulfonic acid
LAC: Lectin affinity chromatography
nanoLC-MS/MS: Capillary liquid chromatography combined with tandem mass spectrometry
PBS: Phosphate buffered saline
PHA-L: *Phaseolus vulgaris* leucoagglutinin
PTM’s: Posttranslational modifications
SDS: Sodium dodecyl sulfate
SDS-PAGE: Sodium dodecyl sulfate polyacrylamide gel electrophoresis
TFA: Trifluoroacetic acid
Tris/HCl: Tris(hydroxymethyl)aminomethane hydrochloride
VVL: *Vicia villosa* lectin

## References

[1] Karavitakis M, Winkler MH, Abel PD, Hazell S, Ahmed HU (2011). Focal therapy for prostate cancer: opportunities and uncertainties. Discov Med.

[2] Schiess R, Wollscheid B, Aebersold R (2009). Targeted proteomic strategy for clinical biomarker discovery. Mol Oncol.

[3] Laidler P, Dulińska J, Mrozicki S (2007). Does the inhibition of c-myc expression mediate the anti-tumor activity of PPAR’s ligands in prostate cancer cell lines?. Arch Biochem Biophys.

[4] Haab BB (2012). Using lectins in biomarker research: addressing the limitations of sensitivity and availability. Proteomics Clin Appl.

[5] Novotny MV, Alley WR, Mann BF (2013). Analytical glycobiology at high sensitivity: current approaches and directions. Glycoconj J.

[6] Novotny MV, Alley WR (2013). Recent trends in analytical and structural glycobiology. Curr Opin Chem Biol.

[7] Varki A, Kannagi R, Toole BP, Varki A, Cummings RD, Esko JD (2009). Glycosylation changes in cancer. Essentials of glycobiology.

[8] Yen TY, Macher BA (2006). Determination of glycosylation sites and disulfide bond structures using LC/ESI-MS/MS analysis. Methods Enzymol.

[9] Laidler P, Dulińska J, Lekka M, Lekki J (2005). Expression of prostate specific membrane antigen in androgen-independent prostate cancer cell line PC-3. Arch Biochem Biophys.

[10] Dulińska J, Gil D, Zagajewski J, Hartwich J, Bodzioch M, Dembińska-Kieć A, Langmann T, Schmitz G, Laidler P (2005). Different effect of beta-carotene on proliferation of prostate cancer cells. Biochim Biophys Acta.

[11] Laemmli UK (1970). Cleavage of structural proteins turing the assembly of the head bacteriophage T4. Nature.

[12] Nilsson S, Wetterhall M, Bergquist J, Nyholm L, Markides KE (2001). A simple and robust conductive graphite coating for sheath less electrospray emitters used in capillary electrophoresis/mass spectrometry. Rapid Commun Mass Spectrom.

[13] Losfeld ME, Leroy A, Coddeville B, Carpentier M, Mazurier J, Legrand D (2011). *N*-glycosylation influences the structure and self-association abilities of recombinant nucleolin. FEBS J.

[14] Hoja-Łukowicz D, Przybyło M, Pocheć E, Drabik A, Silberring J, Kremser M, Schadendorf D, Laidler P, Lityńska A (2009). The new face of nucleolin in human melanoma. Cancer Immunol Immunother.

[15] Masiuk M, Urasinska E, Domagala W (2007). Intranuclear nucleolin distribution during cell cycle progression in human invasive ductal breast carcinomas in relation to estrogen receptor status. Anticancer Res.

[16] Mourmouras V, Cevenini G, Cosci E, Epistolato MC, Biagioli M, Barbagli L, Luzi P, Mannucci S, Miracco C (2009). Nucleolin protein expression in cutaneous melanocytic lesions. J Cutan Pathol.

[17] Watanabe T, Tsuge H, Imagawa T, Kise D, Hirano K, Beppu M, Takahashi A, Yamaguchi K, Fujiki H, Suganuma M (2010). Nucleolin as cell surface receptor for tumor necrosis factor-alpha inducing protein: a carcinogenic factor of Helicobacter pylori. J Cancer Res Clin Oncol.

[18] Watanabe T, Hirano K, Takahashi A, Yamaguchi K, Beppu M, Fujiki H, Suganuma M (2010). Nucleolin on the cell surface as a new molecular target for gastric cancer treatment. Biol Pharm Bull.

[19] Reyes-Reyes EM, Akiyama SK (2008). Cell-surface nucleolin is a signal transducing P-selectin binding protein for human colon carcinoma cells. Exp Cell Res.

[20] Hwang do W, Ko HY, Lee JH, Kang H, Ryu SH, Song IC, Lee DS, Kim S (2010). A nucleolin-targeted multimodal nanoparticle imaging probe for tracking cancer cells using an aptamer. J Nucl Med.

[21] Zhao J, Meyerkord CL, Du Y, Khuri FR, Fu H (2011). 14-3-3 proteins as potential therapeutic targets. Semin Cell Dev Biol.

[22] Obsil T, Obsilova V (2011). Structural basis of 14-3-3 protein functions. Semin Cell Dev Biol.

[23] Aitken A (2011). Post-translational modification of 14-3-3 isoforms and regulation of cellular function. Semin Cell Dev Biol.

[24] Tzivion G, Gupta VS, Kaplun L, Balan V (2006). 14-3-3 proteins as potential oncogenes. Semin Cancer Biol.

[25] Freeman AK, Morrison DK (2011). 14-3-3 proteins: diverse functions in cell proliferation and cancer progression. Semin Cell Dev Biol.

[26] Gardino AK, Yaffe MB (2011). 14-3-3 proteins as signaling integration points for cell cycle control and apoptosis. Semin Cell Dev Biol.

[27] Bustos DM (2012). The role of protein disorder in the 14-3-3 interaction network. Mol BioSyst.

[28] Kleppe R, Martinez A, Døskeland SO, Haavik J (2011). The 14-3-3 proteins in regulation of cellular metabolism. Semin Cell Dev Biol.

[29] Silberring J, Ciborowski P (2010). Biomarker discovery and clinical proteomics. Trends Analyt Chem.

